# Establishment of Functioning Human Corneal Endothelial Cell Line with High Growth Potential

**DOI:** 10.1371/journal.pone.0029677

**Published:** 2012-01-19

**Authors:** Tadashi Yokoi, Yuko Seko, Tae Yokoi, Hatsune Makino, Shin Hatou, Masakazu Yamada, Tohru Kiyono, Akihiro Umezawa, Hiroshi Nishina, Noriyuki Azuma

**Affiliations:** 1 Department of Ophthalomology, National Center for Child Health and Development, Tokyo, Japan; 2 Department of Developmental and Regenerative Biology, Medical Research Institute, Tokyo Medical and Dental University, Bunkyo-ku Tokyo, Japan; 3 Department of Reproductive Biology, National Research Institute for Child Health and Development, Tokyo, Japan; 4 Department of Ophthalmology, Keio University School of Medicine, Tokyo, Japan; 5 Division for Vision Research, National Institute of Sensory Organs, National Tokyo Medical Center, Tokyo, Japan; 6 Division of Virology, National Cancer Center Research Institute, Tokyo, Japan; 7 Sensory Functions Section, Research Institute, National Rehabilitation Center for Persons with Disabilities, Tokyo, Japan; Instituto Butantan, Brazil

## Abstract

Hexagonal-shaped human corneal endothelial cells (HCEC) form a monolayer by adhering tightly through their intercellular adhesion molecules. Located at the posterior corneal surface, they maintain corneal translucency by dehydrating the corneal stroma, mainly through the Na^+^- and K^+^-dependent ATPase (Na^+^/K^+^-ATPase). Because HCEC proliferative activity is low *in vivo*, once HCEC are damaged and their numbers decrease, the cornea begins to show opacity due to overhydration, resulting in loss of vision. HCEC cell cycle arrest occurs at the G1 phase and is partly regulated by cyclin-dependent kinase inhibitors (CKIs) in the Rb pathway (p16-CDK4/CyclinD1-pRb). In this study, we tried to activate proliferation of HCEC by inhibiting CKIs. Retroviral transduction was used to generate two new HCEC lines: transduced human corneal endothelial cell by human papillomavirus type E6/E7 (THCEC (E6/E7)) and transduced human corneal endothelial cell by Cdk4R24C/CyclinD1 (THCEH (Cyclin)). Reverse transcriptase polymerase chain reaction analysis of gene expression revealed little difference between THCEC (E6/E7), THCEH (Cyclin) and non-transduced HCEC, but cell cycle-related genes were up-regulated in THCEC (E6/E7) and THCEH (Cyclin). THCEH (Cyclin) expressed intercellular molecules including ZO-1 and N-cadherin and showed similar Na^+^/K^+^-ATPase pump function to HCEC, which was not demonstrated in THCEC (E6/E7). This study shows that HCEC cell cycle activation can be achieved by inhibiting CKIs even while maintaining critical pump function and morphology.

## Introduction

Human corneal endothelial cells (HCEC) are hexagonal in shape and form a fragile monolayer lying posterior to the surface of the cornea. These cells maintain corneal transparency by their tight intercellular barrier and perform an ion transport pump function through Na^+^/K^+^-ATPase, which regulates the hydration of the corneal stroma [1], [2]. If HCEC sustain damage, excessive hydration and opacity of the cornea occur, resulting in decreased vision.

Corneal endothelia are believed not to increase in adult humans and in fact gradually decrease by approximately 0.5% per year [3], [4], [5]. Damage, injury or HCEC disease such as Fuchs' corneal dystrophy [6], diabetes [7], trauma [8], cataract surgery [9] or elevation of intraocular pressure [10] does not lead to increased proliferation but rather to an increase in cell size to compensate for the wounded area [11]. Once the cell number falls below 1,000 cells/mm^2^, the monolayer of enlarged HCEC cannot maintain corneal translucency [12] and surgical treatment is required to restore vision.

Penetrating keratoplasty has long been the surgical treatment of choice, involving replacement of a total layer of cornea by donor material. However, it can also result in adverse effects such as astigmatism and severe rejection requiring long term usage of immunosuppressive drugs [13]. Recently, alternative transplantation strategies, including modified posterior lamellar keratoplasty techniques such as deep lamellar endothelial keratoplasty (DLEK) [14], Descemet's stripping with endothelial keratoplasty (DSEK) [15] and Descemet membrane endothelial keratoplasty (DMEK) [16] have been introduced to overcome these problems. Despite these advances, an increasingly aging population requiring corneal transplants and inadequate tissue quality limit the availability of donor corneas, such that alternative ways of preparing endothelial cell monolayers need to be explored.

HCEC were originally believed to be incapable of expanding *in vitro*, but have been successfully isolated and cultured by introducing stimulating agents such as epidermal growth factor, platelet-derived growth factor-BB, bovine pituitary extract and fetal bovine serum [17], [18]. However, the number of cells with proliferative activity and the ability to respond to such agents is relatively low, and much variation in proliferative activity exists between donors of different ages [19], [20]. Thus, there is a requirement to achieve a stable and effective culture of cells in terms of both cell proliferation and physiologic function.

The HCEC cell cycle is mainly regulated by the p53 and pRB pathways, both of which have been inactivated by human papilloma virus (HPV) type 16 E6/E7 to successfully immortalize cells. Kim et al. reported the establishment of an immortalized HCEC line using HPV type 16 E6/E7 on lyophilized human amniotic membrane [21]. However, several studies have reported carcinogenesis of the cell line established by viral oncogenes including HPV type 16 E6/E7 or SV40 large T antigen [22], [23]. Therefore a corneal endothelial cell line developed in this way does not appear to be suitable for the treatment of human corneal diseases. To resolve this problem, we expressed mutant cyclin-dependent kinase (Cdk) 4 and CyclinD1 to inactivate the pRB pathway and generate corneal endothelial cell lines without transducing viral oncogenes.

## Results

**Figure 1:**
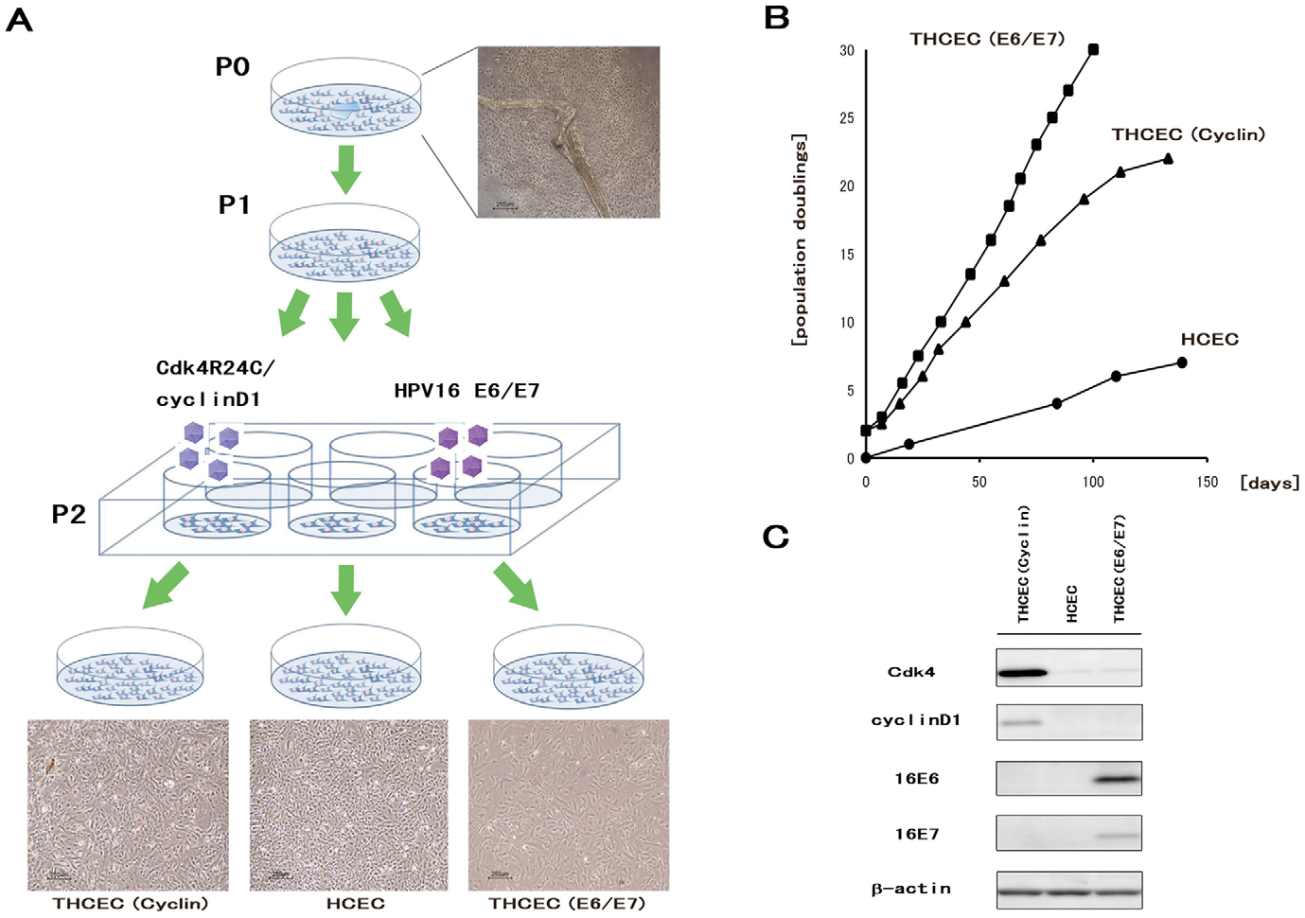
Establishment of THCEC (E6/E7), THCEC (Cyclin) and HCEC. (A) HCEC with Descemet's membrane were placed on Type IV collagen-coated 35 mm cell culture dishes with growth medium (P0). After one passage (P1), retroviral infection was conducted in 6-well cell culture dishes at P2. THCEC (E6/E7) and THCEC (Cyclin) were infected by retroviral vectors carrying HPV16 E6/E7 and both CyclinD1 and Cdk4R24C, respectively. (B) Growth curves of THCEC (E6/E7), THCEC (Cyclin) and HCEC cell lines. THCEC (E6/E7) was immortalized as reported previously, and THCEC (Cyclin) obtained the same proliferative activity as that of THCEC (E6/E7). Transfection was performed on day 0 for THCEC (E6/E7) and THCEC (Cyclin), with population doublings of 2. For HCEC, primary culture commenced on day 0. (C) Western blotting confirmed the expression of the following transgenes: E6 and E7 in THCEC (E6/E7), and CyclinD1 and Cdk4R24C in THCEC (Cyclin).

HCEC with Descemet's membranes were proliferated slowly in a culture dish coated in type IV collagen. After two passages, the cells were transferred into 24-well dishes and transfected with a retroviral vector carrying E6/E7 or mutant Cdk4 and CyclinD1. Three cell lines were successfully generated, as shown in Fig. 1A, with obvious differences in growth (Fig. 1B). Protein expression from the transduced gene was confirmed by western blotting (Fig. 1C). As previously reported [21], THCEC (E6/E7) was immortalized, and THCEC (Cyclin) demonstrated the same proliferative capacity as THCEC (E6/E7), while primary cells grew more slowly even when cultured in 10% fetal bovine serum. These results indicate that induction of mutant Cdk4 and CyclinD1 is sufficient to generate a HCEC line that proliferates at a faster rate than the primary cell line.

**Figure 2:**
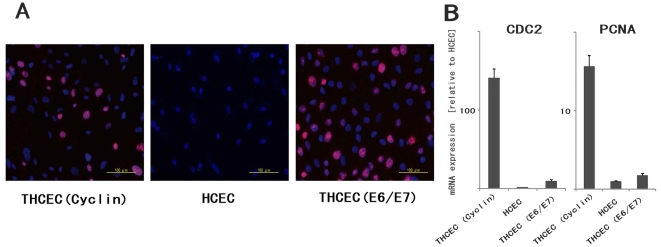
Evaluation of proliferative capacity. (A) Immunohistochemistry of Ki-67 in three cell lines. Positive staining of Ki-67, located in the nucleus, was obviously identified in THCEC (Cyclin) and THCEC (E6/E7), but rarely detected in HCEC. (B) Real-time PCR of downstream genes of cyclinD1 associated with proliferation. Gene expression levels of both CDC2 and PCAN were clearly higher than that of HCEC. The gene expression was much more activated in THCEC (Cyclin) in which the expression of E2F, an upstream transcriptional factor of two genes, was constitutively activated by transduced mutant Cdk4 and CyclinD1.

Proliferation capacity was also confirmed by immunohistochemistry of Ki-67 (Fig. 2A). Expression of downstream genes of CyclinD1 which are associated with cell proliferation was analyzed by real-time polymerase chain reaction (PCR) (Fig. 2B). Positive staining of Ki-67, which is detected in the nucleus, was confirmed in both THCEC (Cyclin) and THCEC (E6/E7). Real-time PCR also revealed that CDC2 and PCNA, target genes of E2F (an upstream transcriptional factor), that are activated by CyclinD1, were up-regulated in THCEC (E6/E7) and especially in THCEC (Cyclin).

**Figure 3:**
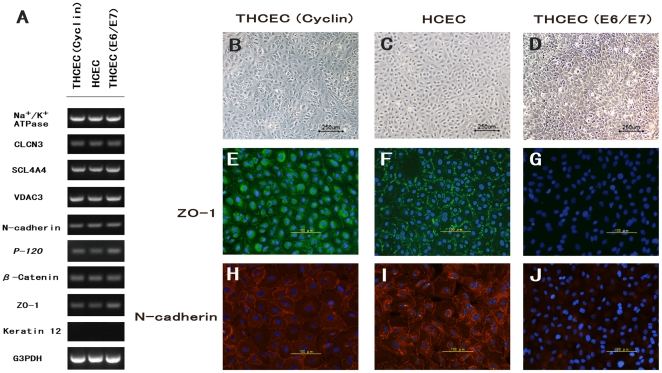
HCEC-associated genes and cytolocalization of junctional components expressed by cell lines. (A) Semi-quantitative reverse transcriptase polymerase chain reaction for HCEC-associated genes. Total RNA was prepared from cultured cells seven days after reaching confluency. No significant difference in mRNA expression was observed between the three cell lines. Compared with phase-contrast micrographs of (B) THCEC (Cyclin), (C) HCEC and (D) THCEC (E6/E7), cytolocalization was examined by immunofluorescence staining of ZO-1 (E, F,G) and N-cadherin (H, I, J). THCEC (E6/E7) did not stain positive for intercellular junctional molecules, while ZO-1 and N-cadherin stained positive at the junction in THCEC (Cyclin) and HCEC.

Expression of genes involved in active transmembrane transporter activity, including Na^+^/K^+^-ATPase, or cell adhesion, including ZO-1 and N-cadherin, were assessed by semi-quantitative reverse transcriptase (RT)-PCR (Fig. 3A). Expression of intercellular adhesion molecules was confirmed by immunohistochemistry (Fig. 3B–J). Semi-quantitative RT-PCR showed that there was no significant difference between the three cell lines regarding the expression of genes associated with several molecules of cell adhesion or of ion transporter channels, which are characteristically expressed by HCEC [21], [24]. This was also confirmed by real-time PCR (data not shown).

ZO-1 and N-cadherin, key HCEC adhesion molecules [24], demonstrated positive staining at the intercellular junction in HCEH (Fig. 3F, I) and THCEC (Cyclin) (Fig. 3E, H), while neither ZO-1 nor N-cadherin was detected in THCEC (E6/E7) despite sufficient cellular density (Fig. 3G, J). Although positive staining of ZO-1 and N-cadherin was observed at the intercellular junction in THCEC (Cyclin), ZO-1 staining also occurred around the nucleus (Fig. 3E), indicating the immature distribution of the ZO-1 protein. In THCEC (Cyclin) and HCEC, hexagonal morphology was identified both by phase-contrast micrography (Fig. 3B, C) and immunocytochemistry, while the structure of hexagonal cell shape was not maintained in THCEC (E6/E7) (Fig. 3D). These data indicate that THCEC (Cyclin) and HCEC, but not THCEC (E6/E7), maintain contact inhibition which is crucial for preserving the monolayer.

**Figure 4:**
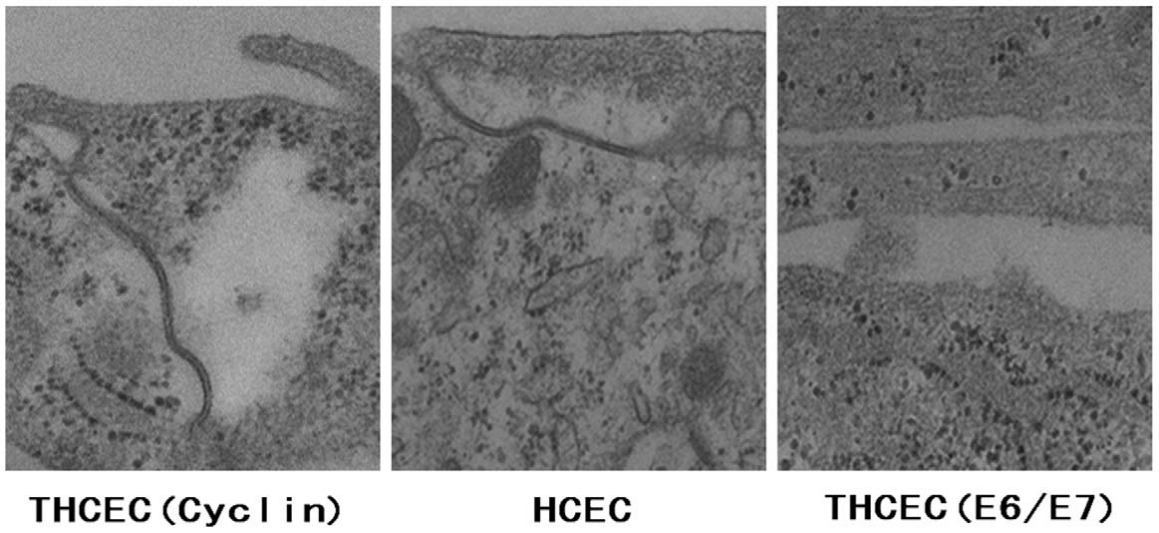
Transmission electron microscopy of cell line intercellular junctions. The junctional complex was detected at the intercellular junction in THCEC (Cyclin) and HCEC. No component of the intercellular junction was found in THCEC (E6/E7), in which cells grew in multilayers without being inhibited by cellular contact (scale bar = 200 nm).

Scanning electron microscopy was performed to reveal detailed information on the cellular junction (Fig. 4). THCEC (Cyclin) and HCEC showed a clear cellular junction including a tight junction, whereas THCEC (E6/E7) grew as a multilayer without forming a cellular junction, which confirms the immunohistochemistry result.

**Figure 5:**
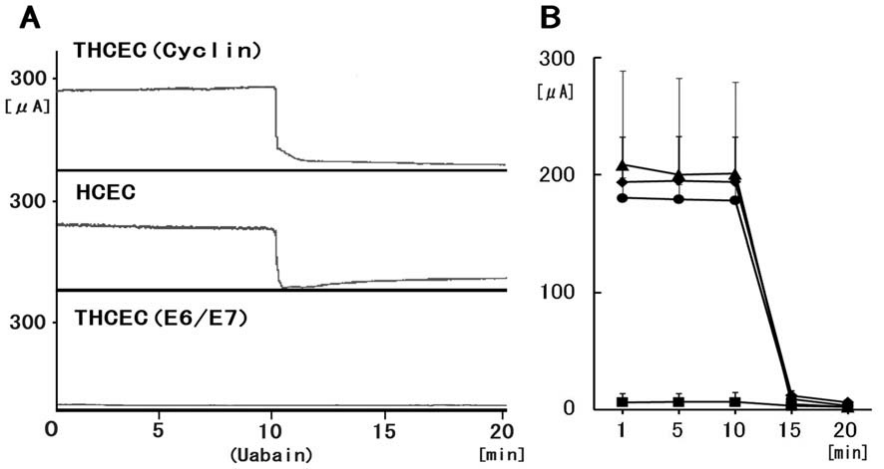
The pump function of cell lines. Short-circuit currents representing Na^+^/K+-ATPase activity from corneal cell monolayers on the insert well area of 4.67 cm^2^ were calculated before and after addition of the Na^+^/K+-ATPase inhibitor ouabain. (A) Representative tracings of short-circuit current (*µ*A/well) obtained with cell monolayers of THCEC (Cyclin) (upper panel), HCEC (middle panel) and THCEC (E6/E7) (lower panel). THCEC (Cyclin) possessed equal transport activity to HCEC, whereas no pump function was detected in THCEC (E6/E7). (B) Time-course changes in the average short circuit current of cultured monolayers of cell lines at 1, 5, 10 and 20 min. Data shown are for (▴) THCEC (Cyclin) at PD8, (♦) THCEC (Cyclin) at PD 21, (•) HCEC and (▪) THCEC (E6/E7); all data are expressed as mean±SD of four replicate experiments of each cell line.

Representative traces of circuit current driven by the Na^+^/K^+^-ATPase were of similar shapes in both HCEC and THCEC (Cyclin) (Fig. 5A). These circuit currents maintain corneal translucency and their levels in both cell lines were clearly reduced by the presence of the Na^+^/K^+^-ATPase inhibitor ouabain, which confirms that the origin of the current is Na^+^/K^+^-ATPase. Meanwhile, the pump function in THCEC (Cyclin), detected in both earlier and later passages of cells, was more variable than that in HCEC (Fig. 5B), possibly indicating incomplete Na^+^/K^+^-ATPase activity or the presence of an intercellular barrier that regulates ion permeability. No regular circuit current was detected in THCEC (E6/E7) (Fig. 5A, B), which probably reflects the absence of intercellular adhesion preventing free ion transport across the membrane. This experiment clearly showed that the THCEC (Cyclin) monolayer has similar Na^+^/K^+^-ATPase activity to that of HCEC.

A tumorigenesis assay of nude mice detected no solid tumor in either THCEC (Cyclin) or THCEC (E6/E7), while HeLa cells formed a solid tumor in all mice (Table 1). Since THCEC (Cyclin) has a similar morphology and pump function to HCEC, THCEC (Cyclin) could be suitable for HCEC studies.

**Table 1:** Tumorigenesis assay of cell lines in BALB/C nude mice.

Inoculated cells	Total dose (cell/mouse)	Number of mice (% mortality)	Number of mice with tumor
THCEC (Cyclin)	1.7×10^6^	3(0)	0
THCEC (E6/E7)	1.7×10^6^	3(0)	0
HeLa cells	2.0×10^6^	3(0)	3

## Discussion

THCEC (E6/E7) was shown to achieve immortalization with a highly activated proliferative capacity, as previously described [21]. However, the cell lines did not show normal intercellular contact or normal pump function, probably because contact inhibition in the cell line was not achieved. Meanwhile, THCEC (Cyclin) was demonstrated to have normal physiologic function with a greater proliferative capacity than primary cells, but slightly lower than that of THCEC (E6/E7).

In expanding the cellular life span, E7 has been shown to play a role in the inactivation of pRB, while E6 activates telomerase [25] and accelerates p53 degradation, which induces the Cdk inhibitor p21 [26]. However, little is known about the effector sites of the viral oncogene that may be related to genetic instability of immortalized cells. In the present study, expression of genes specific to HCEC was not drastically different between the three cell lines. However, key proteins including ZO-1 and N-cadherin that are important in forming intercellular contacts were detected, probably because of the unknown influence of viral oncogenes on post-translational modification, posttranslational import or protein stability/degradation.

We recently established genetically stable, non-transformed immortalized ovarian surface epithelium (OSE) cell lines without viral oncogenes by expressing mutant Cdk 4, CyclinD1 and hTERT, based on the hypothesis that inactivation of the pRb pathway and activation of telomerase are sufficient for OSE immortalization [27]. Meanwhile, Rane et al. demonstrated that mutant Cdk 4 (Cdk4R24C) is sufficient to induce carcinogenesis in several other tissues including those of the pancreas, pituitary and brain [28], and Joyce and colleagues showed that HCEC are arrested in the G1 phase and regulated by CKIs, p16INK4a and p21WAF1/Cip1 [29]. Considering the importance of maintaining morphology and physiologic function in HCEC, we only transduced mutant Cdk 4 and CyclinD1, not hTERT, in the present study. We believe that our careful method enabled THCEC (Cyclin) to form a fragile and regularly arranged monolayer complete with physiologic function.

Although THCEC (Cyclin) has similar characteristics to primary HCEC, immunohistochemistry and the Ussing chamber assay also highlighted the differences between the cells. ZO-1 protein was expressed around the nucleus of THCEC (Cyclin) but not in primary cells. Since semi-quantitative PCR detected almost the same level of mRNA expression between the cell lines, staining around the nucleus in THCEC (Cyclin) probably reflects an error in posttranslational import of ZO-1 protein. The Ussing chamber assay detected a similar pump function between THCEC (Cyclin) and primary cells, but the current in THCEC (Cyclin) was more variable than that of the primary cells, which might have been caused by reduced Na^+^/K^+^-ATPase activity, immature intercellular adhesion allowing irregular intercellular ion transport or differences in cellular density.

Cells established by a retrovirus carry a potential risk of promoting carcinogenesis [30], and direct transplantation to humans of cell sheets composed of such cells may lead to complex problems. Recently, to resolve this problem, several studies have reported the establishment of untransfected corneal endothelial cell lines [31], [32], [33], which are the most ideal cell lines for the treatment of human corneal disease. Meanwhile, alternative bioengineering approaches, including lipofection of p27kip1 siRNA [34], proteomics technology analyzing the difference between younger and older HCEC [35] and drug usage of promyelocytic leukemia zinc finger protein, a cell cycle transcriptional repressor and negative regulator [36], have also been introduced. The present findings support the idea that targeting the interaction between p16INK4a and Cdk4 using such methods is a promising strategy to generate HCEC with sufficient proliferative capacity and physiologic function.

## Materials and Methods

### Isolation and cell culture of human corneal cells

#### Ethics Statement

A cornea was excised from the surgically enucleated eye of a 2-year-old infant undergoing therapy for retinoblastoma, with the approval (approval number, #156) of the Ethics Committee of the National Institute for Child and Health Development, Tokyo, Japan. Signed informed consent was obtained from the donor's parents, and the surgical specimens were irreversibly de-identified. All experiments handling human cells and tissues were performed in line with the tenets of the Declaration of Helsinki.

The corneal piece, which was grossly normal with no pathological lesions, was cut 1.5 mm from the corneal limbus, avoiding contamination of the trabecular meshwork tissue. HCEC with Descemet's membrane were stripped from the posterior surface of the corneal tissue with sterile surgical forceps under a dissecting microscope. They were cut into two pieces and cultured in a cell culture dish covered with Type IV collagen in a growth medium (GM); Dulbecco's modified Eagle's medium (DMEM)/Nutrient mixture F12 (1∶1) with high glucose supplemented with 10% fetal bovine serum, insulin-transferrin-selenium and MEM-NEAA (Gibco, Auckland, NZ). Cells were subcultured after reaching confluency by treating with trypsin/EDTA and seeded at a density of 5×10^5^ cells/well in 6-well dishes.

### Viral vector construction and viral transduction

Lentiviral vector plasmids, CSII-CMV-cyclin D1 and -CDK4R24C were constructed by recombination using the Gateway system (Invitrogen, Carlsbad, CA) as described previously [37]. Briefly, cDNAs of human cyclinD1 and a mutant form of Cdk4 (Cdk4R24C: an inhibitor resistant form of Cdk4, generously provided by Dr Hara) were recombined with a lentiviral vector, CSII-CMV-RfA (a gift from Dr Miyoshi), by LR reaction to create a Gateway expression plasmid (Invitrogen) according to the manufacturer's instructions.

Previous work has described the production of recombinant lentiviruses with the vesicular stomatitis virus G glycoprotein [37], the recombinant retrovirus vector plasmid, pCLXSN-16E6E7 encoding HPV16 E6/E7 (16E6E7) [38] and recombinant retroviruses [39]. Following the addition of recombinant viral fluid to cells seeded in 24-well dishes in the presence of 4 µg/ml polybrene, the cells were infected by the viruses. Stably transduced cells with an expanded life span were designated transduced human corneal endothelial cell by E6/E7 (THCEC (E6/E7)) and transduced human corneal endothelial cell by Cdk4R24C/cyclinD1 (THCEH (Cyclin)).

### Culture of transfected cell lines and growth curve

When the cultures reached subconfluence, the cells were harvested with 0.25% trypsin and 1 mM EDTA, collected into tubes, and centrifuged. The cells were counted using a cell viability analyzer (Vi-CELL Cell Viability Analyzer, Beckman Coulter, Brea, CA), and population doubling (PD) was calculated. The pellets were suspended in growth medium, and the cells were passaged at a density of 5×10^5^ cells/well in a 100-mm dish. The original cells were regarded as PD 2 (day 0).

### Western blot analysis

Western blotting was conducted as described previously [40]. Antibodies against Cdk4 (ser473; Cell Signaling Technology, Danvers, MA), CyclinD1 (clone G124-326; BD Biosciences, Franklin Lakes, NJ), β-actin (sc-1616; Santa Cruz Biotechnology, Santa Cruz, CA) were used as probes, and horseradish peroxidase-conjugated anti-mouse, anti-rabbit (Jackson Immunoresearch Laboratories, West Grove, PA) or anti-goat (sc-2033; Santa Cruz Biotechnology, Santa Cruz, CA) immunoglobulins were employed as secondary antibodies.

### Immunocytochemistry

Cell lines were grown on Type IV collagen-coated glass dishes 14 days after reaching confluency and were fixed with 4% formaldehyde (pH 7.0) for 15 min at room temperature. Cell lines were then rehydrated in phosphate buffered saline (PBS), incubated with 0.2% Triton X-100 for 15 min and rinsed three times with PBS for 5 min each. After incubation with 2% BSA to block nonspecific staining for 30 min, cell lines were incubated with anti-ZO-1 (1∶50; sc-8146; Santa Cruz Biotechnology, Santa Cruz, CA), anti-N-cadherin (1∶50; sc-7939; Santa Cruz Biotechnology) and anti-Ki67 (1∶100; ab15580; Abcam, Cambridge, UK) for 16 h at 4°C. After three washes with PBS, cell lines were incubated with the secondary antibody for 60 min, followed by counterstaining with 4′,6-diamidino-2-phenylindole (1∶200; sc-3598; Santa Cruz Biotechnology) for 10 min.

### Semi-quantitative RT-PCR

Total RNA was extracted from 1×10^6^ cultured HCEC using the RNeasy Plus mini-kitH (Qiagen, Germantown/Gaithersburg, MA) according to the manufacturer's instructions and quantified by absorption at 260 nm. Total RNA was then reverse-transcribed into cDNA using Superscript III Reverse Transcriptase (Invitrogen, Carlsbad, CA) with oligo random hexamers. cDNAs of each component were amplified by PCR using specific primers and DNA polymerase. The reaction was first incubated at 95°C for 10 min, followed by 39 cycles at 98°C for 30 s, 58°C for 30 s and 74°C for 30 s. PCR primers are listed in Table 2.

**Table 2:** Oligonucleotide sequences for RT-PCR.

Name	Sequence	Size (bp)	Accession Number
Collagen type IV	F: 5′-GGC ACC TGC CAC TAC TAC GC-3′	472	NM_001845
	R: 5′-TCA CCA GGA GGT AGC CGA T-3′		
Keratin 12	F: 5′-GAT GCT AAT GCT GAG CTC GA-3′	393	NM_000223
	R: 5′-ACC TGC CCT ACA GCT TTG TA-3		
VDAC3	F: 5′-TGA CTC TTG ATA CCA TAT TTG TAC CG-3′	482	NM_001135694
	R: 5′-TCA ATT TGA CTC CTG GTC GAA-3′		
CLCN3	F: 5′-AGA AAG GCA TAG ACG GAT CAA-3′	204	NM_001829
	R: 5′-GGT TGT ACC ACA ACG CAC TAA-3′		
SLC4A4	F: 5′-GTT CAG ATG AAT GGG GAT ACGC	697	NM_001136260
	R: 5′-CGA GCA TAA ACA CAA AGC GTA A-3′		
Na^+^/K^+^-ATPase	F: 5′-CCC AGG ACT CAT GGT TTT TC-3′	482	NM_000702
	R: 5′-GGA GCA AAG CTG ACC TGA AC-3′		
N-cadherin	F: 5′-CAA CTT GCC AGA AAA CTC CAG G-3′	205	NM_ 001792
	R: 5′-ATG AAA CCG GGC TAT CTG CTC-3′		
β-catenin	F: 5′-TAC CTC CCA AGT CCT GTA TGA G-3′	180	NM _001904
	R: 5′-TGA GCA GCA TCA AAC TGT GTA G-3′		
*P-120*	F: 5′-CCC CAG GAT CAC AGT CAC CT-3′	144	NM_001085467
	R: 5′-CCG AGT GGT CCC ATC ATC TG-3′		
ZO-1	F: 5′-AGT CCC TTA CCT TTC GCC TGA-3′	180	NM_003257
	R: 5′-TCT CTT AGC ATT ATG TGA GCT GC-3′		
GAPDH	F: 5′-GCT CAG ACA CCA TGG GGA AGG T-3′	474	NM_002046
	R: 5′-GTG GTG CAG GAG GCA TTG CTG A-3′		
PCNA	F: 5′- GCGTGAACCTCACCAGTATGT-3′	76	NM_002592
	R: 5′- TCTTCGGCCCTTAGTGTAATGAT-3′		
CDC2	F: 5′- GGATGTGCTTATGCAGGATTCC-3′	100	NM_001786
	R: 5′- CATGTACTGACCAGGAGGGATAG-3′		

VDAC3: voltage-dependent anion channel 3, CLCN3: chloride channel protein 3, SLC4A4: sodium bicarbonate cotransporter membrane.

### Quantitative real-time RT-PCR

Total RNA extraction and reverse transcription into cDNA was carried out as above. Each quantitative real-time RT-PCR for target genes, including Cell Division Cycle 2 (*CDC2*) and proliferating cell nuclear antigen (*PCNA*), was performed using the Chromo4 real time detection system (Bio-Rad, Hercules, CA). For a 20 ml PCR, the cDNA template was mixed with the primers to final concentrations of 200 nM and 10 µl of SsoFast EvaGreen Supermix (BIO-RAD), respectively. The reaction was first incubated at 95°C for 10 min, followed by 45 cycles at 95°C for 10 s, 57°C for 15 s, and 72°C for 20 s.

### Transmission Electron Microscopy

Cell lines cultured on Type IV collagen-coated dishes were fixed in HEPES buffered 2% glutaraldehyde and subsequently post-fixed in 2% osmium tetroxide for 3 h on ice. Specimens were then dehydrated in graded ethanol and embedded in the epoxy resin. Ultrathin sections were obtained by ultramicrotomy and stained with uranyl acetate for 10 min and modified Sato's lead solution for 5 min then submitted to TEM observation (JEM-2000EX, JEOL).

### Measurement of pump function

The pump function of confluent monolayers of HCEC was measured using an Ussing chamber as described previously [41]. Cells cultured on Snapwell inserts coated with Type IV collagen were placed in the Ussing chamber EM-CSYS-2 (Physiologic Instruments, San Diego, CA) with the endothelial cell surface side in contact with one chamber and the Snapwell membrane side in contact with another chamber. The chambers were carefully filled with Krebs-Ringer bicarbonate (120.7 mM NaCl, 24 mM NaHCO_3_, 4.6 mM KCl, 0.5 mM MgCl_2_, 0.7 mM Na_2_HPO_4_, 1.5 mM NaH_2_PO_4_ and 10 mM glucose bubbled with a mixture of 5% CO_2_, 7% O_2_ and 88% N_2_ to pH 7.4). The chambers were maintained at 37°C using an attached heater.

The short-circuit current was sensed by narrow polyethylene tubes positioned close to either side of the Snapwell, filled with 3 M KCl and 4% agar gel and connected to silver electrodes. These electrodes were connected to the computer through the Ussing system VCC-MC2 (Physiologic Instruments) and an iWorx 118 Research Grade Recorder (iWorx Systems, Dover, NH), and the short-circuit current was recorded by Labscribe2 Software for Research (iWorx). After the short-circuit current had reached a steady state, ouabain (final concentration, 1 mM) was added to the chamber, and the short-circuit current was re-measured. The pump function attributable to Na^+^/K^+^-ATPase activity was calculated as the difference in short-circuit current measured before and after the addition of ouabain.

### Tumorigenesis assay

Cells were harvested by Trypsin/EDTA treatment, collected into tubes, and centrifuged, and the pellets were suspended in DMEM. The same volume of Basement Membrane Matrix (BD Biosciences) was added to the cell suspension. Cells (1.7×10^6^) of THCEC (Cyclin) and THCEC (E6/E7) were inoculated subcutaneously into dorsal flanks of each of three Balb/c nu/nu mice (CREA, Japan) for 60 days. A total of 2.0×10^6^ HeLa cells per mouse were used as positive controls. The skin of dorsal flanks of inoculated mice was surgically opened and the tumorigenic status was examined.
